# Elite Athletes’ In-event Competitive Anxiety Responses and Psychological Skills Usage under Differing Conditions

**DOI:** 10.3389/fpsyg.2017.02280

**Published:** 2017-12-22

**Authors:** John E. Hagan, Dietmar Pollmann, Thomas Schack

**Affiliations:** ^1^“Neurocognition and Action – Biomechanics” – Research Group, Faculty of Psychology and Sport Sciences, Bielefeld University, Bielefeld, Germany; ^2^Center of Excellence “Cognitive Interaction Technology”, Bielefeld, Germany

**Keywords:** interpretation, culture, anxiety, frequency, psychological skills

## Abstract

**Background and Purpose:** Even though the assessment of competitive anxiety responses (intensity, interpretation, and frequency) using the time-to-event paradigm has gained much attention, literature on the account of these same experiences in-event and their corresponding psychological skills adopted under differing conditions is limited. This is a follow up investigation to establish the extent to which associated anxiety responses are stable or dynamic and whether this pattern could be related to reported psychological skills under low or high stressful conditions across gender.

**Methods:** Twenty-three high level (*N* = 13 males and 10 females) Ghanaian Table Tennis players provided data through completion of modified versions of Competitive State Anxiety Inventory-2, incorporated with directional and frequency of intrusion scales and the Test of Performance Strategies inventory during breaks within competitive fixtures.

**Results:** MANCOVAs (gender × stress condition) with follow-up analyses revealed no significant interactions and no main effect for gender but significant main effects were realized for all anxiety dimensions and psychological skills for only the second factor. Specifically, the intensity and frequency of cognitive and somatic state anxiety symptoms increased and were interpreted as debilitative under the high stress condition, although self-confidence and other array of psychological skills were highly displayed under the same stressful condition.

**Conclusion:** Findings highlight the dynamic characteristics of in-event associated anxiety responses and ineffectiveness of deployed psychological skills regardless of gender. These perhaps show the exceptionality of affective experiences in an African setting, suggesting a culturally diversified approach to psychological skills application, if desirable effects are to be attained.

## Introduction

People react to environmental stressors in different ways, particularly in terms of emotional experiences like multidimensional state anxiety that unfold over time (Cerin et al., 2000, 2001; Hanton et al., 2008; Mellalieu et al., 2009). From this anxiety perspective, the intensity sub-components of cognitive and somatic anxiety respond differently to the prevalent stressors within the environment (Martens et al., 1990). In competitive sport, athletes atypically report arousal and other somatic and emotional changes during competition (Gould et al., 1993b). While often uncomfortable, and in some cases debilitating, these emotional reactions appear to be essential for optimal response to competitive demands (Hanton and Jones, 1997; Hanton and Connaughton, 2002; Fletcher et al., 2006).

However, research investigations have questioned whether increases in competitive state anxiety experiences are actually detrimental to performance (Parfitt and Hardy, 1987; Jones and Cale, 1989). Consequently, Jones (1991, 1995) proposed a directional dimension of competition-related cognitions that are interpreted as having either positive or negative effects on performance along a facilitative-debilitative continuum. Research findings show that intra-individual differences in anxiety states are not always reflected by changes in the intensity of response. Rather, between-group alterations in the direction of anxiety have rather been shown when the intensity reaction of each group is similar (Jones et al., 1994; Eubank and Collins, 1997; Eubank and Collins, 2000). Additionally, the direction of anxiety appears to play a vital role in terms of how anxiety operates to influence behavior, especially across time (Eubank et al., 1997; Hanton et al., 2004b).

Sequel to the directional perspective, some researchers have advocated for the inclusion of the frequency component of state anxiety reactions. This component refers to the amount of time spent attending to symptoms experienced concerning competition (Swain and Jones, 1993). Although state anxiety intensity and frequency are closely related, these constructs could be examined and regarded as distinct dimensions that independently add to affective experiences (Diener et al., 1991; Kardum, 1999). Studies have shown that individuals are able to report frequency symptoms of emotional experiences with more precision and less recall bias than intensity symptoms of the same emotional experience (e.g., Thomas and Diener, 1990; Diener et al., 1991). There seems to be a distinct gap of temporal based research on the frequency component of anxiety responses (Cerin et al., 2000; Woodman and Hardy, 2001). Additionally the concurrent measurement of these anxiety dimensions in one research design still remains sparse. Indeed, if valence of anxiety can vary over time, then measuring it from within and between individual patterns of state anxiety reactions across different circumstances other than a single snapshot assessment would be appropriate. Even though there has been considerable research on pre-competitive state anxiety responses involving elite athletes, scholarly information on in-event changes of these experiences over differing conditions across similar populations is limited (Eubank and Collins, 2000; Neil et al., 2012).

To manage these emotional experiences, athletes ought to have a repertoire of different coping options available to them. These athletes can then build on what they believe to be the most effective strategy depending on the competitive circumstances (Eubank and Collins, 1997, 2000). According to Eubank and co-worker, most sports have the potential to put competitors under considerable stress due to pressure to perform. Similarly, the impact of discrete positive and negative emotions like anxiety within any encounter as well as potential options and resources for coping (e.g., preventing) and future prospects (e.g., affecting positive change) may also predict subsequent performance outcomes (Lazarus, 1991, 2000).

One moderating variable that has been shown to discriminate against competitive state anxiety responses is the use of basic psychological skills (Mellalieu et al., 2006). For instance, self-confidence is reported to influence anxiety interpretation by offering individuals protection against the debilitating effects of anxiety (Hardy et al., 1996; Mellalieu et al., 2006). Hanton et al. (2004a) explored the psychological skills that underpin competitive anxiety interpretation and found that elite performers reported using cognitive confidence management strategies including mental rehearsal, thought stopping, and positive self-talk against the debilitating interpretations of competitive anxiety. Fletcher and Hanton (2001) also proved that performers who reported greater usage of relaxation strategies, experienced lower levels of anxiety and interpreted symptoms as more beneficial to performance than their comparison groups. From intervention perspective, support has been found for the use of both individual skills (imagery; Hale and Whitehouse, 1998; Page et al., 1999) and multimodal psychological skill packages (goal setting, imagery, and self-talk; Hanton and Jones, 1999; Mamassis and Doganis, 2004) in changing interpretations of symptoms in elite and non-elite populations. Evidence from other studies further suggest that highly successful athletes have better concentration, high self-confidence, have more task-oriented and positive thoughts, use more positive imagery to visualize success, and have lower levels of anxiety. These athletes’ have more developed plans for competition and performance evaluation (Gould et al., 1993a,b; Weinberg and Gould, 2003). Collectively, these findings suggest that elite athletes utilize more psychological skills to enhance self-confidence and protection against the debilitating effects of stressful situations. Therefore, understanding whether changes in athletes’ affective states through the use of basic psychological skills during competitive events would require a more dynamic approach (Anshel et al., 2001; cf. Holt and Hogg, 2002; Poczwardowski and Conroy, 2002) or a trait-centered perspective (Krohne and Hindel, 1988; cf. Crocker and Isaak, 1997; Yoo, 2001) or a combination of both paradigms (e.g., Bouchard et al., 2004; Anshel and Si, 2008) to regulate athletes’ emotional instability would be worthwhile.

From practitioners’ viewpoint, showing gender differences toward athletic stress at elite level has practical implications for sport performance. This is because males and females may experience different types and level of stressors that may require different psychological skills for successful resolution (Anshel et al., 2009). Previous general anxiety literature indicates that whereas males showed no changes in cognitive anxiety and self-confidence levels during pre-competition, females instead experienced gradual elevation in scores of somatic anxiety intensity and a decline in self-confidence (Martens et al., 1990; Jones et al., 1991; Krane and Williams, 1994; Russell et al., 1998). Even though these findings have been challenged in a recent study (Hagan et al., 2017a), as to whether these changes would manifest in-event at elite level is yet to be documented. Hagan and associates found that elite females were less cognitively anxious, interpreted somatic symptoms as more facilitative and were more stable, 7 days before competitive fixtures compared to their male counterparts. Cultural differences were cited as potential reasons for these gender findings. Other coping studies have also shown that gender differentiates the selection of coping strategies in general psychology literature, yet there is little attention from researchers in relation to competitive athletes (Anshel et al., 2009). Although there is an established link between psychological skills and competitive stress, caution ought to be taken when comparing outcomes between male and female athletes (Mahoney et al., 1987; Mahoney, 1989). According to Mahoney and associates, subjects in previous studies were chosen from a wide range of sporting activities and because of the myriad of sporting activities that these athletes participated in, the differences reported may be due to environmental and team climate rather than to gender *per se*. While assessing gender differences among elite athletes is relatively sparse (Woodman and Hardy, 2003), determining the links between the degree of stressful events on elite athletes’ state anxiety dimensions and the subsequent use of related psychological skills as a function of gender is apparently unknown.

Given that cultural differences were cited as potential reasons for the variance in a sample of elite athletes from our previous study (Hagan et al., 2017a), socio-cultural perspective suggests that patterns of emotional experience emanate from different shared norms, social behaviors, and values (Basabe et al., 2000; Mesquita and Markus, 2004). Hence, psychological skills to regulate them may also not be universal across cultures (Ekman and Davidson, 1994). Instead, these skills may be enmeshed in different cultures through learned experiences from diverse societies.

Therefore, exploring a sport like table tennis in a culturally diversified setting would be significant. Table tennis evokes a lot of emotional and cognitive loads on performers due its task complexity and situational demands. Like other fast paced and reactive sports with discontinuous tasks of short duration, table tennis has a very short response window often dictated by high speed of the ball (Raab et al., 2005). These elements force players to use advance cues to decide what appropriate responses are required and what movement patterns ought to be calibrated to optimize performance (Mann et al., 2007). Table tennis players usually compete against their opponents not only through physical actions but also through the display of emotions. These players try to identify the emotions of their opponents and match histories to improve their own control of the competitive situation. As such, they hide or misrepresent their own emotions to influence the judgments that their opponents make as well as use emotional expression as a tool to influence events in order to meet expectations (Sève et al., 2005, 2007). Therefore, any emotional instability may cause habitual technical faults (performance errors) that may affect subsequent match outcomes.

Consequently, the aim of this present study was to establish whether competitive state anxiety dimensions would be stable or dynamic and whether any reported pattern might be related to deployed psychological skills under different stressful situations during competitive fixtures. The following tentative hypotheses were proposed. First, no significant differences in competitive state anxiety intensity would be observed across low and high stress conditions. Second, a more positive competitive state anxiety interpretation would be experienced under low stress condition than under high stress condition. Third, less frequency of intrusions for competitive state anxiety would manifest under low stress condition than under high stress condition. Lastly, a greater array of psychological skills would be displayed under high stress condition than under low stress condition. These aforementioned hypotheses were tested across gender after removing the effects of age and years of experience.

## Materials and Methods

### Participants

The study was approved by the ethics committee [Institutional Review Board (IRB)] of Bielefeld University after adhering to the ethical standards of the sixth revision of the Helsinki Declaration. Twenty-three elite Ghanaian table-tennis players (*N* = 13, males; *N* = 10, females) were purposively sampled from the initial 90 who participated in study 1 (pre-competition) as a follow-up investigation. Data were collected under two different stress-related conditions. The athletes’ competitive status was determined by their international standing. Specifically, participants’ elite status was determined by players who had received honors and represented Ghana at international events approved by International Table Tennis Federation (ITTF; e.g., African/World Championships or Commonwealth Games, etc.), a procedure used in similar studies (e.g., Hanton et al., 2005). Players’ ages ranged from 21 to 33 years. All participants had been actively competing in this sport for 8–12 years, trained on the average three to four times a week and were competing in the national league for their respective clubs. All participants signed a written consent form prior to the commencement of the data collection.

### Instrumentation

#### Modified Competitive State Anxiety Inventory-2 (CSAI-2)

The Competitive State Anxiety Inventory-2 (CSAI-2) (Martens et al., 1990) assesses competitive anxiety and self-confidence. The inventory was modified to include scales for direction (Jones and Swain, 1992) and frequency (Swain and Jones, 1993) dimensions. This measuring instrument assesses symptoms via 27 items, 9 per construct. The intensity scale is anchored on a 1 (not at all) to 4 (very much so) while the direction scale is measured on a -3 (very debilitative: negative) to +3 (very facilitative: positive); 0 = unimportant interpretation scale. The frequency scale is also assessed on a 1 (never) to 7 (all of the time) scale. Therefore, intensity scores range from 9 to 36, direction scores from -27 to +27, and frequency scores from 9 to 63 across each construct. Internal reliability values (Cronbach alpha) for the intensity scale range from 0.79 to 0.90 (Martens et al., 1990), the direction scale from 0.72 to 0.89 (Jones and Hanton, 1996; Hanton et al., 2000), and the frequency scale from 0.70 to 0.93 (Thomas et al., 2004) respectively. Values (Cronbach coefficient alpha) in the current study range from 0.80 to 0.83 for intensity, 0.79 to 0.84 for direction, and 0.81 to 0.84 for frequency scales. These values are appropriate and consistent with previous research.

#### Test of Performance Strategies (TOPS)

The Test of Performance Strategies (TOPS) (Thomas et al., 1999) examines athletes’ use of activation, relaxation, imagery, goal-setting, self-talk, automaticity, emotional control, and negative thinking/attentional control during practice and competition. There are four items in each subscale. Based on previous research findings, a clear rationale was present for examining relaxation, goal-setting, imagery, and self-talk skills during competition (Jones and Hanton, 1996; Hale and Whitehouse, 1998; Hanton and Jones, 1999). Consequently, the other subscales were not used in the present study. Items during competition, for example, include relaxation, “I am able to relax if I get too nervous at competition”; and for goal-setting, “I set personal performance goals for a competition”; for imagery, “I imagine my competitive routines before I do them at competition”; and for self-talk, “I talk positively to myself to get the most out of competition”. Participants ranked the frequency of each item on a scale anchored by 1 (“never”) to 5 (“always”), with total psychological skills scores ranging from 4 to 20. Previous reported Cronbach alpha coefficients are 0.80 for relaxation, 0.78 for goal-setting, 0.79 for imagery, and 0.80 for self-talk (Thomas et al., 1999). Internal reliability (alpha coefficient values) in the current study are as follows; self-talk, 0.95; goal setting, 0.94; imagery, 0.94; and relaxation, 0.94, values that are high and acceptable.

### Procedure

The 23 participants were monitored under low and high stressful conditions during competition at a national championship dubbed “Top Ten” that was organized purposely to select players for the 2015 African Championship in Congo. The momentary assessments (ecological sampling method) of selected anxiety and coping variables were taken during intervals between matches and during extended breaks during the competition. Specifically, Stressful status was determined by the potential disproportion between demand and response competence, where failure to meet the required demand has important consequences. Competition is one such situational stressor; therefore, the magnitude of situation was categorized from the preliminary rounds as “low stressful” whereas the advance phases were considered “high stressful”. Competitive stakes were assumed to be very high because previous studies have proven that emotions are probably more intense when high esteemed goals are at stake (Lazarus, 1999; Uphill and Jones, 2007). These phases in competition usually have a higher demand and importance. In line with Martens et al. (1990) theory of competitive anxiety, the importance of outcome was used as the distinguishing variable between each condition. The advanced phases of the competitive events were selected as being more stressful than the preliminary stages. The completion of the instruments was triggered by a random signal used during the breaks between matches during the competition.

An initial brief introductory session was held for participants to explain the format for the completion of the instruments. Participants were required to note their intensity, interpretation, and frequency of cognitive, somatic symptoms, and self-confidence experienced during these periods and the corresponding psychological skills implemented in order to deal with increases in their emotional episodes during competition.

### Data Analysis

To reduce the error variance due to possible confounding variables, four multivariate analysis of covariance (MANCOVA) repeated measures were conducted on the data (Tabachnick and Fidell, 2007; Field, 2009). Within the MANCOVA model, competitive state anxiety [three sub-constructs (cognitive, somatic, self-confidence) across three dimensions (intensity, direction, frequency) and four psychological skills (self-talk, goal-setting, imagery, relaxation)] served as the dependent variables whereas gender (male, female) acted as an independent variable. As continuous variables, age and years of experiences acted as covariates. A separate follow up univariate analyses of covariance (ANCOVA) were conducted to compare the means for each of the dependent variables across gender and stress condition to determine where significant differences existed through further *post hoc* analyses. Partial eta squares values were calculated and interpreted as small effect (0.20), medium or moderate effect (0.50), and large effect (0.80; Tabachnick and Fidell, 2007; Field, 2009). Before using MANCOVA, data prescreening procedures were conducted to examine the accurateness of the data and statistical assumptions of homogeneous regression coefficients (assumption of common slope), homogeneity, and multicollinearity of variances were all assessed. Sphericity assumption was also assessed by means of Mauchly’s test for the within-subject repeated measure analyses, and whenever the test was violated, appropriate technical modifications were done using the Greenhouse–Geisser test (Tabachnick and Fidell, 2007; Field, 2009). Descriptive statistics (means, standard deviations, standard errors) between the study variables were also calculated. All data analyses and related procedures were conducted using the Statistical Package for Social Sciences [(SPSS) version 22.0 for Windows].

## Results

### Preliminary Analyses

The distribution of the data satisfied the assumptions for univariate and multivariate analyses (Tabachnick and Fidell, 2007; Field, 2009). No missing cases and univariate or multivariate outliers within each dependent variable (Mahalanobis distance test) were identified. In addition, assumptions of normality, linearity, multicollinearity, and singularity were deemed satisfactory. However, equality of covariance matrices assumption, although satisfactory at the univariate level (Levene’s test and F_max_ ratios), was violated in some cases at the multivariate level (Box’s test). Hence, Pillai’s trace was selected as the multivariate test statistic due to its robustness over test violations (Tabachnick and Fidell, 2007; Field, 2009). Descriptive statistics (means and standard error) for all variables are shown in **Table 1**.

**Table 1:** Adjusted and unadjusted means for anxiety, self-confidence, and psychological skills collapsed across gender.

Variable	Stress condition	Adjusted mean (SE)	Unadjusted mean (SD)
CA-I	Low	21.93 (0.85)	21.58 (5.23)
	High	27.03 (0.90)	17.42 (5.49)
SA-I	Low	17.44 (1.04)	17.18 (5.08)
	High	24.44 (1.04)	24.18 (5.08)
SC-I	Low	29.98 (0.97)	29.90 (4.71)
	High	33.98 (0.97)	33.90 (4.71)
CA-D	Low	8.46 (1.08)	8.43 (5.49)
	High	5.46 (1.08)	5.43 (5.49)
SA-D	Low	5.00 (1.14)	-5.05 (4.95)
	High	-1.99 (1.14)	-1.94 (4.95)
SC-D	Low	20.71 (0.91)	20.71 (4.28)
	High	15.71 (0.91)	15.71 (4.28)
CA-F	Low	30.69 (1.35)	30.29 (7.60)
	High	33.69 (1.35)	33.29 (7.60)
SA-F	Low	25.03 (1.96)	24.55 (9.31)
	High	31.03 (1.96)	30.55 (9.31)
SC-F	Low	47.22 (2.19)	47.11 (9.71)
	High	52.22 (2.19)	52.11 (9.71)
ST	Low	16.21 (0.72)	16.20 (3.04)
	High	18.34 (0.52)	18.31 (2.18)
GS	Low	15.89 (0.57)	15.86 (2.67)
	High	17.66 (0.51)	17.65 (2.42)
IM	Low	15.58 (0.75)	15.55 (3.31)
	High	17.97 (0.62)	17.92 (2.70)
RL	Low	13.41 (0.71)	13.34 (3.69)
	High	10.47 (0.67)	10.42 (3.52)

*SE, standard error; standard deviation; CA, cognitive anxiety; SA, somatic anxiety; SC, self-confidence; I, intensity; D, directional interpretation; F, frequency of intrusions.*

No interaction effects were noted across all analyses (*p* > 0.05) indicating that any change-over-time patterns were consistent (or parallel) across the gender categorization. Data were subsequently condensed across males and females for the stress condition analysis (**Table 1**). The identification of significant main effects for gender or stress condition main effects was followed with one-way ANCOVAs testing for gender or within-subject repeated measure effects for stress condition. A follow-up *t*-test with the Bonferroni correction factor was applied where necessary (Tabachnick and Fidell, 2007; Field, 2009).

### Main Analyses

#### Anxiety Intensity Dimension

The covariates (age and years of experience) were significantly related to the anxiety intensity, age, Pillai’s trace = 0.458, *F*(3,15) = 4.218, *p* = 0.02 and years of experience, Pillai’s trace = 0.335, *F*(3,15) = 2.519, *p* = 0.03.

**FIGURE 1:**
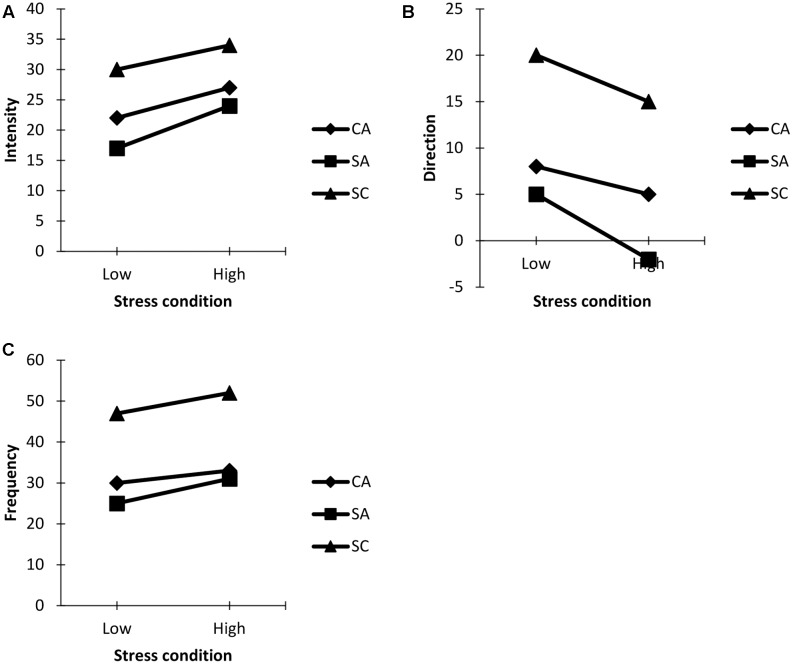
**(A)** State anxiety and self-confidence intensity pattern. **(B)** State anxiety and self-confidence direction pattern. **(C)** State anxiety and self-confidence frequency pattern. **(A–C)** Competitive state anxiety and self-confidence responses across low and high stress conditions.

After controlling for the effects of the covariates, there was no significant main effect of gender, Pillai’s trace = 0.249, *F*(3,15) = 1.655, *p* > 0.05. However, significant multivariate main effects were noted for the stress condition, Pillai’s trace = 0.462, *F*(1,17) = 14.618, *p* < 0.001, $\eta_{p}^{2}$ = 0.46. A follow-up within-subject (stress condition) ANCOVAs revealed changes for cognitive state anxiety, *F*(1,1000) = 17.628, *p* < 0.001, $\eta_{p}^{2}$ = 0.55, somatic state anxiety, *F*(1,1000) = 13.534, *p* < 0.001, $\eta_{p}^{2}$ = 0.34 and self-confidence, *F*(1,1000) = 14.804, *p* < 0.001, $\eta_{p}^{2}$ = 0.44 respectively. An inspection of the corrected *t*-test showed an increase in both cognitive and somatic state anxiety intensity symptoms from low stress to high stress condition although self-confidence symptoms improved from low to high stress condition during the same competition period (**Figure 1A**).

#### Anxiety Direction Dimension

There was no evidence of effects for the covariates (age and years of experience) on anxiety direction, age, Pillai’s trace = 0.026, *F*(3,15) = 0.133, *p* > 0.05 and years of experience, Pillai’s trace = 0.075, *F*(3,15) = 0.404, *p* > 0.05.

No significant main effect of gender, Pillai’s trace = 0.166, *F*(3,15) = 0.992, *p* > 0.05 was realized. However, significant multivariate main effects were noted for the stress condition, Pillai’s trace = 0.448, *F*(1,17) = 11.426, *p* < 0.001, $\eta_{p}^{2}$ = 0.59, with follow-up within-subject (stress condition) ANCOVAs indicating changes for cognitive state anxiety, *F*(1,1000) = 12.821, *p* < 0.001, $\eta_{p}^{2}$ = 0.64, somatic state anxiety, *F*(1,1000) = 14.032, *p* < 0.001, $\eta_{p}^{2}$ = 0.38 and self-confidence, *F*(1,1000) = 13.204, *p* < 0.001, $\eta_{p}^{2}$ = 0.44 respectively. Specifically, cognitive state anxiety facilitative interpretation decreased during the high stress condition whereas an initial somatic state anxiety facilitative interpretation under the low stress condition changed to a debilitative interpretation under the high stress condition. Also, an initial positive self-confidence interpretation display under low stress condition decreased under the high stress condition during the competition period (**Figure 1B**).

#### Anxiety Frequency Dimension

There was no evidence of effects for the covariates (age and years of experience) on anxiety frequency, age, Pillai’s trace = 0.253, *F*(3,15) = 1.691, *p* > 0.05 and years of experience, Pillai’s trace = 0.129, *F*(3,15) = 0.741, *p* > 0.05.

No significant main effect of gender, Pillai’s trace = 0.038, *F*(3,15) = 0.196, *p* > 0.05 was revealed. However, significant multivariate main effects were revealed for the stress condition, Pillai’s trace = 0.673, *F*(1,17) = 13.227, *p* < 0.001, $\eta_{p}^{2}$ = 0.46, with follow-up within-subject (stress condition) ANCOVAs noting changes for cognitive state anxiety, *F*(1,1000) = 5.041, *p* < 0.001, $\eta_{p}^{2}$ = 0.43, somatic state anxiety, *F*(1,1000) = 6.984, *p* < 0.001, $\eta_{p}^{2}$ = 0.53 and self-confidence, *F*(1,1000) = 6.254, *p* < 0.001, $\eta_{p}^{2}$ = 0.49 respectively. Specifically, frequency of both cognitive and somatic state anxiety symptoms increased under the high stress condition after an initial low intrusions reportage under the low stress condition. Self-confidence frequency appeared to improve from the low to high stress condition during the same competition period (**Figure 1C**).

### Psychological Skills

There was no evidence of effects for the covariates (age and years of experience) on anxiety direction, age, Pillai’s trace = 0.070, *F*(4,14) = 0.264, *p* > 0.05 and years of experience, Pillai’s trace = 0.087, *F*(4,14) = 0.334, *p* > 0.05.

**FIGURE 2:**
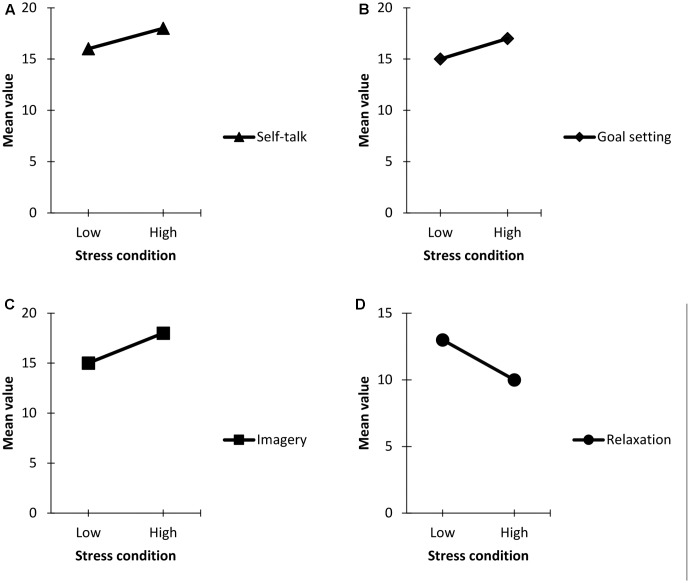
**(A)** Self-talk pattern; **(B)** goal setting pattern; **(C)** imagery pattern; **(D)** relaxation pattern. **(A–D)** Reported psychological skills pattern across low and high stress conditions.

After controlling for the effects of the covariates, there was no significant main effect of gender, Pillai’s trace = 0.409, *F*(4,14) = 2.423, *p* > 0.05. However, significant multivariate main effects were revealed for the stress condition, Pillai’s trace = 0.793, *F*(4,14) = 15.331, *p* < 0.001, $\eta_{p}^{2}$ = 0.99. A follow-up within-subject (stress condition) ANCOVAs indicated differences for self-talk, *F*(1,1000) = 6.150, *p* < 0.001, $\eta_{p}^{2}$ = 0.65, goal-setting, *F*(1,1000) = 6.585, *p* < 0.001, $\eta_{p}^{2}$ = 0.68, imagery, *F*(1,1000) = 4.287, *p* = 0.05, $\eta_{p}^{2}$ = 0.50 and relaxation, *F*(1,1000) = 50.064, *p* < 0.001, $\eta_{p}^{2}$ = 0.99 respectively. An inspection of the corrected *t*-test showed self-talk, goal-setting, and imagery slightly improved with much higher reported mean values under high stress condition compared to the low stress condition whereas reported relaxation skills usage decreased with a much lower mean value under high stress condition compared to the low stress condition (**Figures 2A–D**).

## Discussion

A major strength of this study was the use of elite table tennis players competing in an ecologically valid field setting, which to some authors, is suggested to be the most attractive environment for the study of competitive anxiety (Jones and Hardy, 1990; Pensgaard and Ursin, 1998). Additionally, it is the first quantitative temporal design that examines multidimensional competitive state anxiety responses and reported corresponding psychological skills across gender under different stressful conditions during competitive fixtures. Despite the important roles of competitive state anxiety and psychological skills at elite level, empirical research has generally ignored potential connections between the two variables during competitive fixtures.

Although the concepts of multidimensional anxiety dimensions, self-confidence and psychological skills could be best described as a function of varied stressful conditions, the extent to which no succinct differences manifested across gender in this current study is surprising. Findings from this study contradict the commonly held belief that gender differentiates multidimensional anxiety experiences, self-confidence (Jones and Cale, 1989; Donzelli et al., 1990; Swain and Jones, 1993) as well as the selection of coping behaviors (Hoar et al., 2006). Results further illustrate that situational demands (stress condition) impacted athletes’ associated anxiety symptoms and self-confidence by the fluctuations shown during the competitive fixtures, a finding that is supported by other studies (Krane et al., 1994; Jones, 1995; Butt et al., 2003). Specifically, elite table tennis players experienced increases in both cognitive and somatic anxiety as well as self-confidence intensity and frequency as the situation became more stressful whereas interpretation responses of the same emotional variables were perceived to be more debilitative during matches under the high stress condition.

Some dimensions from cultural psychology, in part, cannot be ruled out as possible reasons that may have accounted for the no variance across gender on the associated anxiety symptoms highlighted earlier on. According to the (Hofstede’s, 1991) masculinity-femininity continuum, Ghana ranks moderately on the masculinity scale, this possibly explains why elite athletes in this study were expressive with internal reactions [mental reactions (cognitive) and physiological (somatic) symptoms] and other possible behavioral reactions regardless of their gender. These findings, according to Basabe and associates, may lower the general well-being of these athletes because of the high frequency of associated anxiety exhibited during the competition (Basabe et al., 2000).

Somatization literature further indicates that, in many societies of the world, the body, and mind are connected in the display of emotional distress irrespective of gender (Dzokoto, 2010). The base rates of these emotional expressions have been found to vary within and across cultures, with non-western cultures associated with more reports of physical symptoms in psychologically distressed persons during stressful situations (Kirmayer et al., 1998).

Again, cultures with strong uncertainty avoidance (e.g., Ghana) tend to experience high levels of anxiety and other negative emotions (e.g., anger and fear). These societies are viewed as aggressive, compulsive and stressful, reinforcing the frequency of emotional experiences exhibited across gender in the current study (Hofstede, 1991; Arrindell et al., 1997).

Our results further show that elite table tennis players experienced varied anxiety symptoms across the dimensions due to situational demands (stress condition), suggesting that on-going actions during matches do not evoke the same tone of emotional experiences under differing stressful situations (Sève et al., 2007). Evidence of this is shown by the high symptoms reported by performers across the anxiety dimensions under the high stressful situations. To Beck (1993), stressful individuals develop a “cognitive set,” that is, they construct a situation that allows them to make a series of appraisals of the external situation (i.e., risks, costs) and react in a particular way. Table tennis players in this study, possibly made sense of unfolding events of their matches histories, in terms of strokes played, points won and lost, different interpretations, awareness about opponents, as well as their concerns and worries. These elements of competitive interactions might have predicted the specific emotional coloration shown by the players in the current study (Sève et al., 2002, 2003, 2007). We also assume that the debilitative interpretations of both cognitive and somatic anxiety in the midst of high self-confidence display could be attributed to controllable factors like unusual technical errors (stroke and movement related problems). These negative interpretations which occurred during the high stress condition were possibly due to high valued goals at stake, suggesting that mastery of techniques (automaticity) and other physical preparations ought to complement the implementation of psychological interventions.

Additionally, the no gender differences reported regarding the use of selected psychological skills across the two different stress conditions challenge the commonly held contention that gender differentiates the selection of coping strategies (Goyen and Anshel, 1998; Hammermeister and Burton, 2004; Hoar et al., 2006; Anshel et al., 2009). However, current findings corroborate with few studies (Pensgaard et al., 1999; Lane et al., 2002; Bebetsos and Antoniou, 2003) that found no gender differences in coping with stressful events in sport. From cross-cultural viewpoint, emotion regulation strategies are not universal and gender specific. Therefore, the deployment of psychological skills may therefore be enmeshed in different cultures such that displayed norms may indicate when, how, and which coping strategy should be displayed to combat in any specific situation regardless of gender (Saarni, 1989; Mesquita and Markus, 2004).

We speculate that other strategies like religious coping (an aspect of religion) might have accounted for the no gender differences in the current sample of athletes. Issues about religion are pervasive in most African settings (e.g., Sub-Sahara; Hagan and Schack, 2017) and are believed by most persons as a source for seeking help in order to cope with daily hassles and other life stressful events irrespective of gender. This sort of coping (e.g., use of prayer) is spurred in part by evidence that religion plays an integral role in the entire stress process. This ranges from its influence on ways in which people appraise events (Park and Cohen, 1993), to its influence on ways in which they respond psychologically and physically to those events over the long term (Seybold and Hill, 2001). Many athletes of Sub-Sahara descent have a strong conviction that prayers, for example, contribute to sporting success (Ikulayo, 1989). Prayers are believed to harmonize the mind and body (relaxation), promote emotional control and positive thought processes, and help block inhibitory thoughts through verbal persuasion and imagery. They are not gender specific and are assumed to facilitate long-term behavior change and enhance sport performance (Heap and Aravind, 2002; Ikulayo and Semidara, 2011). They have some functional similarities with psychological skills (e.g., self-talk, goal-setting, imagery, relaxation; Heap and Aravind, 2002; Ikulayo and Semidara, 2011). From research standpoint, religious coping has received little attention in sport (Folkman and Moskowitz, 2004) and warrants empirical investigations to test their efficacy and effectiveness across different cultures (Hagan and Schack, 2017).

One significant issue in coping research is whether psychological skills are stable or dynamic within a specific context (i.e., situation to situation; Dias et al., 2012; Hagan et al., 2017b). Evidence in the present study shows support for the combined influence of both stable and situational factors (e.g., Carver and Scheier, 1994; Rutherford and Endler, 1999; Bouchard et al., 2004; Anshel and Si, 2008). Elite athletes’ psychological skills did influence their reactions in new situations and predicted the selection of psychological skills in response to their stressful situations (Neil et al., 2007, 2009). These athletes consistently varied their cognitive and behavioral efforts (i.e., self-talk, goal setting, imagery, and relaxation) to manage specific demands (i.e., different phases of their competitive fixtures) that were probably appraised as taxing (Lazarus and Folkman, 1984).

However, associated anxiety experiences were unrelated to the reported use of psychological skills, with anxious elite athletes still reporting usage of different psychological skills. This emphasizes the fact that specificity between type of psychological skill and anxiety dimension is crucial because reported skills did not impact on the high level and frequency of reported cognitive and somatic anxiety symptoms under the high stressful conditions. It is possible that elite athletes might have tried other types of psychological skills that were not captured in this study to cope with their anxiety. Alternatively, it is also possible that the continuous use of reported psychological skills under the high stress condition led to ponderings and greater attention to maladaptive cognitions, mirroring the high level and frequency of competitive anxiety exhibited. This partly explains the decrease usage of relaxation skills, a situation that can potentially cause distortions in the neuromuscular mechanisms of these athletes and impact negatively on subsequent performance. The anxiety interpretation perceived under the high stress condition further supports the maladaptive coping claim mentioned earlier. Again, if these athletes do not have requisite psychological skills to deal effectively with their stressful situations, they may experience performance slumps and negative affect (Madden, 1995; Giacobbi and Weinberg, 2000).

The use of psychological skills can also be more or less effective when encountering a demanding situation like playing crucial competitions. Therefore, the mere deployment of a psychological skill or a combination of them, does not necessarily guarantee its effectiveness (Folkman and Moskowitz, 2004). However, a causal link cannot be ascertained within this present design because it was not possible to determine if these anxious athletes were merely reporting or using or discarding these strategies after they proved ineffective in regulating their anxiety. Additional longitudinal research is therefore required to gain better understanding of coping effectiveness and its relationship with competitive anxiety to further guide appropriate interventions (Nicholls and Polman, 2007). According to Nicholls et al. (2005), this is one research area that is considered difficult and not fully understood.

Although our small and homogeneous sample limits the generalizability of these findings to other groups of elite table tennis players, we contend that the studied variables were very important and the use of ecological momentary assessment warranted full examination. The momentary assessment approach in the participants’ natural sport environment overcomes some of the weaknesses of retrospective designs that dominate research literature by improving accuracy of self-reports (Brandstätter, 1983; Hormuth, 1986; Nicholls et al., 2005). In retrospective designs, participants are likely to report how they would normally behave, compared to ecological momentary assessment where participants are more likely to report how they actually feel in a particular situation (Smith et al., 1999). This approach, likened to diary process method or experience sampling method, facilitates analysis of changes in stress appraisals and coping in real time or close to real time occurrence (Lazarus and Folkman, 1984; Lazarus, 1999; Conner et al., 2009). Therefore, coping studies in sport with the continuous use of these assessment approaches may disclose relevant information for researchers and applied practitioners. Future research could use ecological momentary assessment to examine coping effectiveness, gender differences, and performance related variables (Nicholls et al., 2005).

In this study, consideration was given to quantitative measures of multidimensional anxiety dimensions (intensity, direction, and frequency) and related psychological skills in differing stressful conditions across gender. Our findings enhance theoretical understanding of anxiety perceived as debilitative by elite table tennis players in some critical situations. These players were unable to deploy effective psychological skills to combat their emotional experiences (Jones, 1995; Biddle and Ntoumanis, 2000). Therefore, elite players’ debilitative anxiety was related to their inability to effectively regulate these negative emotions. The strategies were dysfunctional during the high stressful situations. The findings indicate loss of situational control and question sport psychologists and coaches who deliver conventional psychological skills in an attempt to manage athletes’ performance related anxiety (Carver et al., 1989). Therefore, more time and effort should be spent on developing appropriate counseling and intervention strategies through psychological skills training and stress man‘agement programs (Eubank and Collins, 2000). Such programs could target conditions that promote more facilitative anxiety interpretation based on Jones’s (1995) model and related research findings.

Although the above findings need further replication, cultural explanations provided earlier on open another chapter for cultural sport psychology research on elite performers because culturally displayed norms guide emotional expressions due to the conglomeration of different traditions, beliefs, and behaviors (Mesquita and Markus, 2004). Further, given that sport psychology literature involving competitive athletes is sparse (McCreary et al., 2006), providing additional knowledge on emotional experiences like competitive anxiety and associated culturally diversified coping applications as a function of gender, and other personal and situational variables to optimize sport performance would be worthwhile.

## Author Contributions

JH conceived the idea of this paper, collected the data, imputed and run statistical analyses, and produced the first draft of the manuscript. DP structured the methodological section of the manuscript (data collection procedures, checked the general statistical analyses) and corrected the first version of the manuscript. TS was restructure the general ideas for the paper, responsible for the final version of the manuscript, did the ethical clearance, and approved of funding for the data collection.

## Conflict of Interest Statement

The authors declare that the research was conducted in the absence of any commercial or financial relationships that could be construed as a potential conflict of interest.
